# How to culturally adapt the pulmonary rehabilitation programme for people living with COPD in Sri Lanka: a qualitative study

**DOI:** 10.1136/bmjresp-2024-002407

**Published:** 2025-07-21

**Authors:** Chamilya Perera, Akila Jayamaha, Mark Orme, Thamara Dilhani Amarasekara, Zainab Yusuf, Amy Barradell, James Manifield, Andy Barton, Ravini Karunathilake, Amitha Fernando, Savithri Wasundara Wimalasekera, Sally J Singh

**Affiliations:** 1 Department of Respiratory Science, University of Leicester, Leicester, UK; 2 Faculty of Nursing, KAATSU International University, Colombo, Western Province, Sri Lanka; 3 Centre for Exercise and Rehabilitation Science, NIHR Leicester Biomedical Research Centre-Respiratory, University Hospitals of Leicester NHS Trust, Leicester, UK; 4 Nursing and Midwifery, University of Sri Jayewardenepura, Nugegoda, Sri Lanka; 5 BMJ, London, UK; 6 Central Chest Clinic, Colombo, Sri Lanka; 7 University of Sri Jayewardenepura Faculty of Medical Sciences, Nugegoda, Sri Lanka

**Keywords:** Pulmonary Rehabilitation, Pulmonary Disease, Chronic Obstructive

## Abstract

**Background:**

Pulmonary rehabilitation (PR) is a low-cost, high-impact intervention for people living with chronic obstructive pulmonary disease (COPD). Despite the high prevalence of COPD, there are currently very limited facilities to provide PR in Sri Lanka. The views of people living with COPD, their caregivers and relevant healthcare professionals (HCPs) are essential to develop culturally appropriate PR, acceptable in a Sri Lankan setting.

**Objectives:**

We aimed to explore the lived experiences of key stakeholders on the development and implementation of culturally appropriate PR in Sri Lanka.

**Methodology:**

A qualitative study was conducted at the Central Chest Clinic (CCC), Sri Lanka. Focus group discussions (FGDs) and semistructured interviews (SSIs) were conducted with the three populations: people living with COPD, their caregivers and relevant HCPs. After audio recording, transcribing and translating, the data were analysed using thematic analysis.

**Results:**

Three FGDs comprising 11 adults with COPD (9 males, age range 39–83 years), three FGDs comprising five family caregivers (three females), three FGDs comprising 14 nurses and 12 SSIs with doctors and physiotherapists were conducted, representing diverse ethnic groups. Two overarching themes were generated: ‘PR adaptations’ and ‘Barriers to PR implementation and adherence’. Within ‘PR adaptations’, four subthemes were generated: the educational component of PR, nutritional support, psychological support and the use of music during PR sessions. Under ‘Barriers to PR implementation and adherence’, three subthemes were generated: barriers and issues in participating, need for better medical facilities and difficulty in conducting exercises.

**Conclusion:**

Culturally tailoring PR for people living with COPD in Sri Lanka should include the integration of singing, music and nutritional support, as it may enhance acceptability. Barriers, including a lack of resources to deliver PR, difficulties encountered by patients attending PR sessions and perceived difficulties in performing standardised PR exercises, need to be addressed when developing a culturally appropriate programme in Sri Lanka.

**WHAT IS ALREADY KNOWN ON THIS TOPIC**

- Although pulmonary rehabilitation (PR) is a beneficial intervention for individuals with chronic obstructive pulmonary disease (COPD), availability of services is scarce in low- and middle-income countries, such as Sri Lanka.

**WHAT THIS STUDY ADDS**

- The current study provides insight from Sri Lankan adults with COPD, their caregivers and healthcare professionals into what a culturally appropriate PR programme in Sri Lanka should provide. Specifically, participants suggested the integration of singing, music and nutritional support, as well as adding an educational component to improve acceptability and enhance community engagement and health outcomes.

**HOW THIS STUDY MIGHT AFFECT RESEARCH, PRACTICE OR POLICY**

- Results from this study may be used to culturally adapt PR programmes in Sri Lanka, which, in turn, may help improve uptake and completion.

## Introduction

 Chronic obstructive pulmonary disease (COPD) is the fourth leading cause of death worldwide, corresponding to 5% of all deaths globally, and is projected to be the third leading cause of death by 2030.^1^ Currently, the burden of disease is higher in Asia than in Europe in relation to the total number of deaths and the burden of disease, as estimated in the number of years of life lost and the number of years spent living with disability.^2^ Similar to the prevalence of COPD globally and in Asia, the recent estimate of COPD in Sri Lanka was 10.5%.^3^ COPD results in progressively worsening airflow limitation, causing breathlessness, sleep disruption, physical inactivity and exercise intolerance, and is linked to an increased risk of hospitalisation^4^ and premature death.^5^

The American Thoracic Society/European Respiratory Society Pulmonary Rehabilitation (PR) Statement (2013) defines PR as ‘a comprehensive intervention based on a thorough patient assessment followed by patient-tailored therapies that include, but are not limited to, exercise training, education and behavior change, designed to improve the physical and psychological condition of people with chronic respiratory disease and to promote the long-term adherence to health-enhancing behaviors’.^6^ PR uses multidisciplinary teams to optimise the physical and psychosocial functioning of patients with COPD.^7^ It is considered a low-cost intervention compared with certain pharmacological treatments^8^ and can be implemented in low-resource settings with minimal equipment.^9^ PR is supported by high-quality evidence that improves the quality of life and reduces the burden of symptoms, frequency of hospitalisations and mortality.^10 11^ International guidelines recommend that PR should be routinely offered to patients with COPD with persistent symptoms, limited activity and recurrent exacerbations.^12^

Sri Lanka is a multicultural and multiethnic country where healthcare is provided free of charge by the state. Currently, there is a limited number of PR programmes being delivered in Sri Lanka.^13^ Diverse cultural beliefs, practices and social support systems can shape patients’ perceptions of health and illness, thus likely to influence their engagement in rehabilitation programmes. To address this, it is important to explore the needs of the population in the context of culture and customs. For example, studies conducted among the Māori people^14^ and in the Kyrgyz Republic^15^ identified key culturally acceptable considerations to improve uptake and acceptability of pulmonary rehabilitation.

Accordingly, this study aimed to qualitatively explore the needs and perceptions of people living with COPD, their caregivers and the healthcare professionals (HCPs) to inform the adaptation and implementation of a culturally appropriate PR programme in Sri Lanka.^16^

## Methodology

Qualitative research methods were adopted to conduct semistructured interviews (SSIs) and focus group discussions (FGDs) with HCPs, patients living with COPD and their caregivers. This study was conducted as part of a broader programme of research, Global Recharge Project, regulated by the University of Leicester, UK. This article was structured according to the COREQ (Consolidated criteria for Reporting Qualitative research) guidelines.^17^

## Study setting

The study was conducted in the Central Chest Clinic (CCC) of the National Hospital of Sri Lanka in Colombo, which is the main healthcare setting that provides treatment for patients with COPD from all over the country. Colombo city consists of a diverse population of various ethnic and cultural groups. The conference hall of the clinic (which is quiet and not used routinely for clinical work) was used for conducting the SSIs and FGDs.

## Recruitment of participants

Eligible HCPs were nurses, doctors and physiotherapists with ≥1 year of experience caring for people living with COPD. They were identified by the researchers with the help of HCPs at the CCC. These were selected using convenient sampling to accommodate the availability of HCPs.

Eligible participants were registered patients living with COPD at CCC, their caregivers. Adults aged ≥18 years, diagnosed with COPD by spirometry and clinically confirmed by consultant respiratory physicians confirmed COPD with spirometry based on GOLD (Global Initiative for Chronic Obstructive Lung Disease) criteria with forced expiratory volume in the first second (FEV_1_)/forced vital capacity <0.7, and FEV_1_ <80% predicted and having a Medical Research Council dyspnoea score grade 2 or higher were included in the sample of patients.^16^ Caregivers aged ≥18 years and self-reported to be closely caring for a person living with COPD were eligible and could also be family members. Participants were identified using purposive sampling to ensure the sample represented the multireligious, multiethnic Sri Lankan cultural diversity and gender balance of patients with COPD in Sri Lanka.

Eligible participants were verbally informed about the study by the researchers. After obtaining verbal consent, detailed study information sheets were provided for further information. If they were willing to participate in the study, all consenting participants provided written informed consent.

## Patient and public involvement

As HCPs, our experiences and conversations with people living with COPD have informed the design of the study, including interview guides and recruitment strategies. The present study builds on our clinical experiences by delving into more detail the rehabilitation needs of this population. In line with the principles of patient and public involvement, findings of this work will be used to directly inform patient-centred care; specifically, appropriate pulmonary rehabilitation. Study findings will be disseminated to participants and the public through events at the CCC in Colombo, as well as at national and international conferences.

## Data collection

Data was collected between March–December 2021. FGDs were conducted with patients with COPD and their caregivers. These were scheduled to align with routine clinic visits. FGDs were also conducted with nurses, at times convenient to them, without interfering with their routine work. SSIs were conducted with doctors and physiotherapists again at times convenient to them, without interfering with their routine work.

FGDs and SSIs were conducted by trained, experienced researchers (CP (female, MSc), AJ (male, MSc), TDA (female, PhD)) using discussion and interview guides with a narrative approach. Full versions of the interview topic guides are available in online supplemental file 1. Prior to data collection, an explanation of what PR entails was provided to all participants to allow them to reflect sufficiently on the programme. Questions and prompts were improved and adapted throughout data collection, informed by reflective notes and discussions of transcribed data, which were held regularly with the research team. The majority of SSIs were conducted in English, as participants found it easier to express their ideas and medical terminology in English. Further, all medical records in the clinics are maintained in English among the doctors and physiotherapists. FGDs with nurses and one SSI were conducted in Sinhala language. All data collected was audio-recorded (Sony ICD-UX570F recorder), transcribed verbatim (CP, AJ) and where necessary, translated into English to enable wider cross-national teams to participate in the analysis. Translations were carried out by team members in Sri Lanka fluent in English and Sinhala (CP, AJ). Team members paid attention to conveying the authentic meanings of the discussions and details, such as laughter in the transcripts with the help of the field notes taken during and after the interviews and discussions. Transcripts were anonymised and compared with the interview audio recordings to ensure completeness and accuracy, without being returned to participants for verification.

## Data analysis

Qualitative data were analysed using codebook thematic analysis by following the steps of Braun and Clarke:^18^ (1) Familiarisation with the data (CP, AJ, ZY, TDA, MO); (2) Coding the data, semantic and inductive coding approaches were adopted (CP, ZY, MO); (3) Generating initial themes (CP, ZY, MO); (4) Reviewing and developing themes (AJ, TDA); (5) Refining, defining and naming themes (CP, ZY, MO); and (6) Producing the report (CP, ZY, MO). Microsoft Word and Excel were used for the data management and analysis.

Coding was conducted by investigators (CP) with support from four members of the team (MO, AJ, ZY, TDA). This involved each researcher familiarising themselves with the interview data, by reading and rereading the transcripts while making reflective notes. The data interpretation was facilitated by assigning initial codes or classifications to segments of text, exploring relationships between these classifications and developing core general themes. Participants’ quotes were extracted to provide supporting examples for the themes.

The research team engaged in continuous reflexivity throughout the study process on their related backgrounds and biases to reduce their influences on the data collection and interpretation. To ensure trustworthiness and rigour, the current research study followed the criteria such as multiple data triangulation, credibility through member checking and data transferability.

## Results

Among the 58 adults with COPD invited to the study, 11 consented to participate. Three FGDs were conducted (one with five members and two with three members in each). Characteristics of the individuals with COPD who participated in the study include male (82%), Sinhala (73%) and unemployed (54.5%) participants. Only 27% of them are employed, and most individuals have completed their education up to ordinary level (27.3%). The sample indicates a wide range in duration since diagnosis, as 18.2% have been diagnosed for 4 years, and the rest of the sample varied between 1 and 10 years.

Two FGDs were conducted with five caregivers (one with three, and the other with two members). The compliance among the caregivers was poor due to COVID restrictions, with only 5/15 invited caregivers participating in the study. Characteristics of caregivers consist of the majority of females (60%), and many have completed their education up to a diploma (40%) and ordinary level (O/L) (40%). All the caregivers are close family members to the patient as the sample represents two sons, two daughters and one sister.

A total of 26 healthcare providers took part in the study (92% Sinhala, 65% female, 3–28 years’ professional experience). Three FGDs were conducted with the nurses (two with five, and one with four members) and SSIs were conducted with the doctors and physiotherapists. All the invited HCPs participated in the study.

These measures were essential to obtain reliable data until data saturation was achieved. The duration of interviews lasted 30–40 min, and FGDs lasted between 30 and 60 min. Recruited patients with COPD or caregivers did not have any previous exposure to PR, and HCPs had very limited exposure.

**Figure 1:**
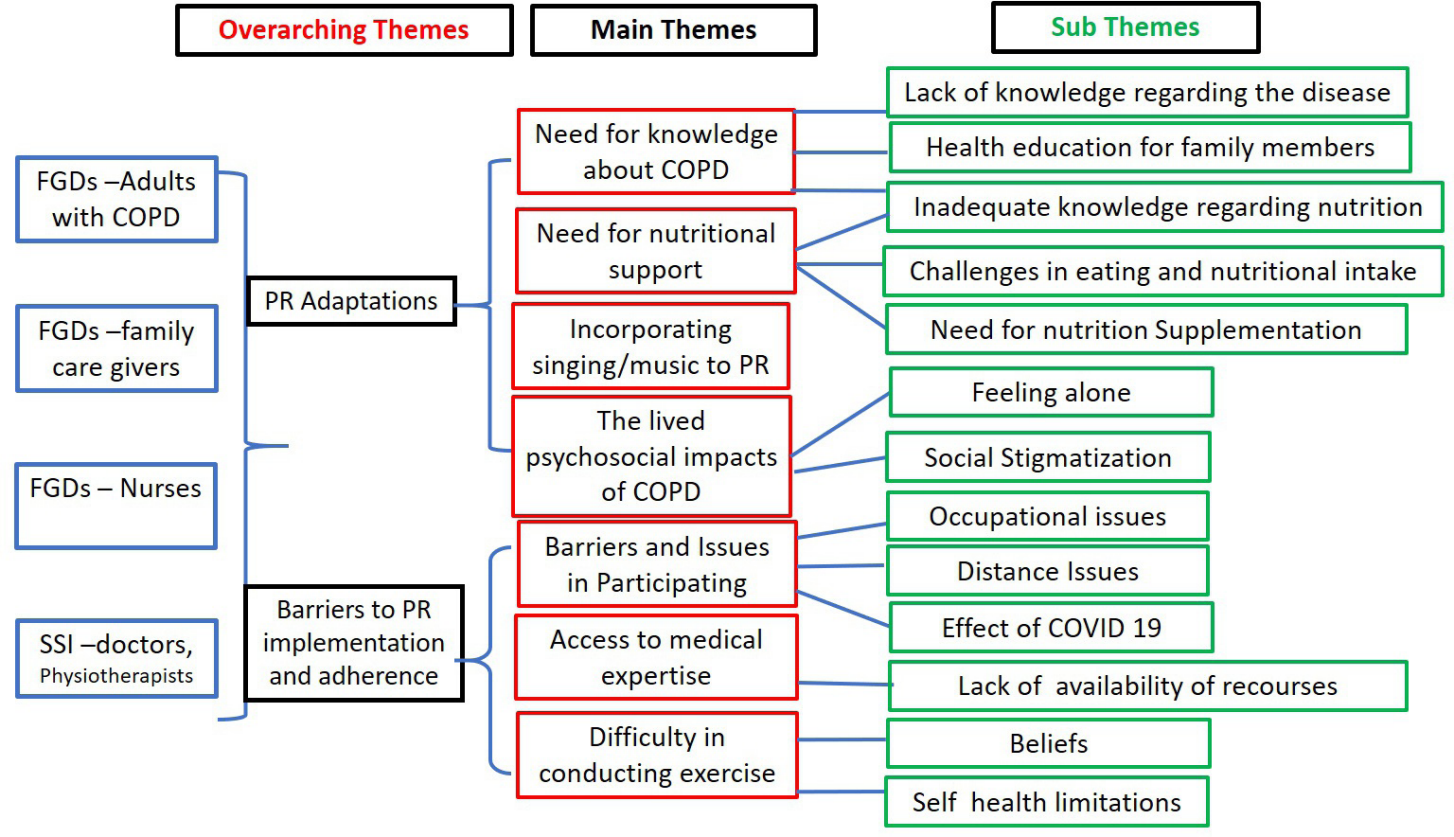
Themes developed by the analysis of semi-structured interviews and focus group discussions with adults with COPD, caregivers and healthcare professionals. COPD, chronic obstructive pulmonary disease; FGD, focus group discussions; PR, pulmonary rehabilitation; SSI, semistructured interview.

Seven themes and 12 subthemes were generated from the data collected. The results are categorised under two overarching concepts to facilitate reporting: the need for PR adaptations and barriers to PR implementation and adherence (figure 1).

## Need for adaptation to pulmonary rehabilitation

As the uptake of PR was low at the CCC, similar to other studies conducted in other centres of the world,^19^ the need for modification and cultural adaptation was identified as a necessity. The adaptations that emerged were as follows: the need for better education about the disease, the need for nutritional support, the need to incorporate singing as a component of PR and the need for psychological support.

The excerpts that emerged from the FGDs and the substantiating evidence obtained from the SSI are explained under each of the main themes below.

### Need for better knowledge about COPD

Many of the discussions conducted with patients and HCPs emphasised it is crucial to improve the level of knowledge regarding the disease amongst patients with COPD, including the importance of symptom management and drug therapy. The education component in PR is essential to increase the knowledge regarding the disease among patients. The increased knowledge regarding their condition may lead to adopting better self-management practices and overall better health outcomes.^20^ Patients were keen to receive more information about their condition as stated below:

Patient FGD (P5) “We are not getting any proper education from here [CCC]’’

The caregivers of patients had many concerns regarding their lack of knowledge about the disease. Their perceptions are expressed as below.

Caregiver FGD (CG2) “We don’t know exactly what is his (her father’s) condition. If he doesn’t take the drugs, he gets breathing difficulty. Therefore, I’m taking him to the clinic once a month.”

The nursing staff who participated in the FGD also voiced the concerns of lack of knowledge of the disease among the patients. Their perceptions are expressed as,

Nurse FGD (N6) “Some people have enough money and feel the need to get well also, but they lack the knowledge and guidance to do so”

The need to provide more information to patients regarding the disease was explained by the doctors who participated in the SSI. Their perceptions are explained by the following excerpts

Doctor SSI (D2) “So if we can arrange some plan, if we can arrange a place that we can refer them to, and we can give some education regarding the disease, and can educate regarding the management options, what are the benefits of those management options, then I think they will adhere to the management plan.”

Almost all the participants believed that the education component is a key point in PR. Further, it emerged that since most of the patients are dependent on their family, it is very important to actively involve their family members in the delivery of PR education sessions.

Nurse FGD (N3) “health education should be expanded to include the relations or family caregivers*”.*

### Need for nutritional support

The topic of nutrition was important for all patients with COPD, caregivers and HCPs. Within this theme, participants discussed the need for nutritional education, nutritional supplementation and the challenges experienced by patients in eating and nutritional intake. Nutrition and food practices appeared to differ depending on cultural and religious beliefs.

HCPs and people with COPD highlighted a lack of awareness around diet and nutrition and emphasised the need to communicate using understandable, non-scientific language.

Patient FGD (P6) “It would be important to know what food we should avoid and what food items we should have more.*”*

Nurse FGD (N4) “…we must use familiar words to them as they will not understand if we say a high protein diet. We have to say what are the food items with protein. Likewise, most of the things we have to explain to them. They do not have very good knowledge regarding food types.”

According to the FGDs conducted with patients with COPD and their caregivers and SSIs with HCPs, they experience challenges due to the intake of food and tolerance. This may lead to an increase in symptoms, breathing difficulties and discomfort.

Patient FGD (P11) “I cannot have egg yolk and cow’s milk. As soon as I eat them it increases my phlegm production and I feel wheezing*”*

Most believe that cold food, according to Ayurveda, stimulates the production of phlegm, sneezing and difficulty in breathing.

Patient FGD (P1) “I cannot eat cold food. As soon as we had eaten them increasing the wheezing feeling.”

Healthcare staff, mainly doctors and nurses, recommended that nutritional supplementation should be provided to people living with COPD. They emphasised the advantages and importance of improving the nutritional status of patients to achieve better outcomes.

Nurses FGD (N4) “…sometimes we have nutrition clinics. According to the BMI, low BMI patients are given this supplementation. But not for all. If we can arrange something even for the people who are very weak, it will be effective.”

Patient FGD (P8) “We would prefer it [nutritional supplementation], especially after exercising. For now, we drink … [Name anonymized - Nutritional supplementation available in local market] only which costs about 1800 rupees [~4.96 USD]. That too we buy once in 2 months. Therefore, if we get something like that it will be helpful.”

### Incorporating singing/music to the PR

Many participants perceived that incorporating singing and music to the Sri Lankan specific PR programme will be beneficial. They were keen to incorporate singing and music as part of PR. This incorporation may lead to the improvement of respiratory functions, enhancing emotional wellbeing and increasing patient engagement in COPD treatments.^21^ Nurses recommended incorporating background music to help patients relax while completing their 256 exercises. Nurses and physiotherapists suggested that the incorporation of musical instruments, to facilitate singing as a part of the PR programme, would motivate the patients.

Nurse FGD (N4) “All the people, no matter whether they are Sinhala, Tamil or Muslim, they all sing *** [name anonymized] some songs very nicely…yes, it is good. Even for the exercises, if we can bring them to an exercise area with background music and if we ask them to do exercises, it will be more effective, I think. They will also be more encouraged to do exercises even at home.*”*

Physiotherapists SSI (P1) “Yes, I do think incorporation of music is good. Even now, I advise patients to bring flutes when they come to the programme. We do use the flute as an exercise technique*”*.

Patient FGD (P5) “Yes, the music helps to relax our minds”.

### The lived psychosocial impacts of COPD

All healthcare providers, patients and caregivers reported various psychosocial impacts of the disease, personal problems and relationship issues (eg, with family). People living with COPD shared their experiences of feeling lonely and the feeling of being rejected by society.

Patient FGD (P3) “Now we are feeling alone. No one cares much”

Patient FGD (P1) “We cannot go anywhere; we cannot do any work alone. We need someone’s support every time. Sometimes I feel very sad about myself*”*.

Further, several participants expressed that they experienced difficulties in performing simple tasks.

Patient FGD (P4) “I’m unable to do even my own simple work sometimes. I can’t walk, wash my clothes or go outside. At that time, I feel really sorry about myself”.

Some people who are living with COPD have had some bad experiences due to the disease, which has adversely affected their social interactions. It had an impact on maintaining relationships with loved ones since most patients believed that COPD was contagious and were afraid of transmitting the disease to their own loved ones. Some expressed their concerns as below.

Patient FGD (P9) “Some people are very afraid of even talking to me. They think that the disease [COPD] will spread to them. So hardly anybody speaks to me*.”*

## Barriers to PR implementation and adherence

### Anticipated barriers and issues in pulmonary rehabilitation participation

Participants expressed the barriers and issues related to participation in PR. Most of these people are having issues in getting leave from their work two times a week to participate in the programme. They described highly expensive travel and transportation costs, limited availability of public transport and the need for a family member to accompany visits to the clinic as difficult logistical problems for most persons with COPD. As data collection was done during the COVID-19 pandemic, participants reported fear of travelling by public transport and were reluctant to make multiple visits to the CCC.

Many of the patients talked about difficulties obtaining public transportation as some areas have poor public transportation services. Most find it difficult to be a standing passenger in public transportation buses, which are crowded during the rush hours. Since most people with COPD are elderly, they need support boarding and alighting from buses and trains. Thus, most of them need an accompanying person to attend regular PR sessions.

Nurses FGD (N3) “Most are elderly people, and they need to be accompanied by their son/daughter due to the long distance of travel. As the accompanying person has to get to work, there is no one to accompany the patients. If there were clinics available near them, they could have come on their own without depending on their children.”

Doctors SSI (D2) “The other thing is distance also, the patients come from far…far away and another extra day they have to come for this and … sometimes when the programme does not end on time patients find it difficult to even find a person to accompany them for the monthly medical clinic and obtain routine medication.*”*

Patient FGD (P5) “We cannot come frequently. It is costing a lot to come. One trip will cost more than Rs. 1000 [3.05 USD]”

One of the main issues expressed by healthcare providers was the loss of wages received from employment when patients with COPD attend PR sessions two times a week. Frequent absenteeism from work would directly impact the income level of the patients. Their perceptions are expressed as follows.

Nurses FGD (N6) “Another problem is that since the patients are employed in professions such as carpentry, mason work etc. they are unable to take leave and come here on the days we allocate for them.”

Doctors SSI (D10) “Most of these patients are daily-wage earning workers. So, if they want to come for some medical advice or some medical programme regularly, then they have to sacrifice their job or some of their daily wages for this. When prioritizing things, they are compelled to give more priority to their work*.”*

### Access to medical expertise

Patients preferred to obtain medical advice from doctors. Since doctors are directly involved with the diagnosis of the disease and planning treatment, and monitoring its course, during the FGDs, participants felt it was important to have a dedicated doctor to support the PR programme. Further, the patients with COPD believed this would be more effective, and adherence to PR thus would be higher.

Patients FGD “Yes, the most important suggestion is that, having an assigned doctor who will check up on us regularly and give advice and prescribe scans if needed. Last time also the Doctor just prescribed some medicines without asking much. He didn’t tell us any details on how long we have to take the medicines whatsoever.”

Doctors SSIs (D8) “As medical officers, we have very little time to spend with the patients. A very small time to explain the patient’s conditions, what the complications are, what the management options and what the benefits of these management options are.”

On the other hand, the lack of time and the non-availability of adequate medical staff contribute to a barrier in the acceptance of the PR programme, thus leading to poor patient outcomes. Most patients were, however, conscious of the time constraints of doctors and how this would need to be considered if the doctors were to be in attendance during the PR sessions.

Lack of medical resources is a significant barrier to providing effective treatment for patients with COPD and also causes a significant delay in the implementation of the PR programme in a clinical setting.^22^ This was expressed by the medical personnel as follows:

Physiotherapist SSI (Ph1) “Mm. Yes, now what I feel is according to the current workload, it is better if we can finish off these sessions by conducting sessions within the morning hours or within the evening hours to facilitate better compliance. For that, we need the support of the medical officers. Because, for now, this programme is overseen by a medical officer. It is a bit difficult to get hold of the doctor. Sometimes he is not available which wastes the patients time too*.*”

Doctor SSI (D1) “As medical officers, we have very limited time to spend with the patients. There is limited Restrictions on the time available to explain the patient’s condition, the anticipated complications, the management options and the benefits of the management options impact patient care and patient compliance.”

### Difficulty in conducting exercises

Some patients with COPD expressed difficulty in doing exercises and had a fear of being involved with the exercise programme. This was because most participants had not engaged with exercises and physical activities actively since the onset of their disease. The fear of relapse, worsening of symptoms and worsening of disability made them reluctant to be involved with the exercises and exercise-related programmes.

Patient FGD (P6) “It’s difficult to do exercises at home.”

Patient FGD (P8) “It’s very difficult to move the extremities, as I haven’t done any hard work for a long time. Now all extremities are locked.”

Caregiver FGD (CG3) “He (the patient) is not doing any activity at home. He cannot do them. He feel very tired even walking a little distance. I’m not sure whether he can do any exercises.”

## Discussion

This study sought to understand the implementation needs for a culturally sensitive PR programme for patients living with COPD in Sri Lanka by appraising the views of people living with COPD, caregivers and HCPs involved with caring for people with COPD. The results indicate two overarching concepts that captured the barriers to PR and the necessary adaptations required to implement PR into the healthcare service. Proposed adaptations to PR referenced the need for an educational component for patients and their family/caregiver, nutritional support, incorporating singing and music to PR and psychological support. Barriers referenced proposed difficulties in participating in a PR programme, availability of medical expertise and difficulty in conducting exercises. The need to develop a culturally appropriate PR programme for the people living with COPD in low- and middle-income (LMIC) settings is an important issue that has not been considerably addressed in the local context. Further, the current study provides a holistic account of the PR treatment experiences as perceived by patients with COPD, their caregivers and healthcare providers in a LMIC. To our knowledge, at the start of this study, there were no PR programmes being conducted in Sri Lanka. Cultural adaptation is not optimised to meet the needs of patients with COPD, their caregivers and healthcare providers.^23^ The current study provides important features that would enhance the acceptability and compliance among all the stakeholders.

Further, this programme would incorporate culturally specific practices related to dietary habits, health maintenance, factors within the community support system that influence the lived experiences of participants. When compared with international guidelines, this adapted programme will be conducted taking into consideration the local influences and inputs obtained from this study. Further, the health education materials and sessions will be delivered in the two main Sri Lankan languages of Sinhala and Tamil, which would enhance the understanding and motivate better engagement among participants. The adapted programme would enhance collaboration among the local HCPs, community and family members, which would create a more supportive environment for the participants. Furthermore, psychological support and nutritional tailoring would provide individualised care that enhances compliance among participants and motivates easily implementable changes into their daily lives.

Disease education is crucial for the positive outcomes of the disease because it supports the adaptation of positive COPD self-management strategies such as pursed lip breathing, early self-recognition of exacerbations and smoking cessation.^24^ Findings of the current study highlighted that the education component is warranted and should illustrate aspects of disease education, the basis of disease management and nutritional management taking into cultural practices, and the benefits of multiple aspects of PR. Proper education built into the PR programme was felt to be an influencing factor for better disease management, improving outcomes and motivating better compliance to PR. Adults with COPD exhibit a heightened susceptibility to disease exacerbation and a diminished likelihood of achieving favourable disease outcomes due to the lack of knowledge and educational resources.^25^ Enhancing knowledge can exert a significant impact on the utilisation of non-pharmacological therapies and the prevention of misconceptions and erroneous practices. Ultimately, it would lead to improved self-management of the disease.^26^

The findings of the current study indicate that patients with COPD, their caregivers perceived that COPD is infectious, similar to tuberculosis (TB), and thus, patients with COPD faced social isolation. Misconceptions that ‘COPD is infectious’ indicate poor health literacy among the people and are not only unique to Sri Lanka.^15 27 28^ Thus, it is essential that health education should address this issue.

Further, consequences such as stigmatisation and discrimination of patients with COPD by society will result in these patients further not seeking appropriate healthcare. This may lead to failure to attend health facilities for fear of being confirmed as having TB disease and being further stigmatised. This creates a vicious circle that results in further delays in diagnosis and contributes to increased morbidity and mortality.

The findings of the current study showed that caregivers play an important active role in disease management and actively engage in the patient’s care and well-being, since all the interviewed caregivers were family members living with the patient with COPD. The capacity of the caregiver to provide appropriate care would get limited as a result of poor knowledge of the disease.^29^ Hence, augmenting the knowledge of the caregiver along with the patients with COPD, via an educational component, would foster improved disease outcomes and patient well-being in our Sri Lankan COPD population. Furthermore, an educational component has been shown to provide an excellent opportunity for caregivers to engage in formal and informal discussions with physiotherapists, medical professionals, nutritionists and any PR trainers.^30^

The need to include nutrition as a main topic in the education component was highlighted by all stakeholders in the current study. Sri Lankan treatment hierarchy includes a combination of Western medicine, Ayurveda medicine and traditional methods such as homeopathy and Unani. Most patients practise a combination of these methods in their daily practice. This will be highly significant since it will highlight the different beliefs and practices prevalent among the general population regarding the disease management and prevention of complications.^31^ Nutrition-based disease management is poised to remain at the forefront of culturally specific beliefs and practices. The influence of these beliefs can manifest in both positive and negative ramifications.^32^ Enhancing nutritional knowledge as well as providing supplementation as suggested by the adults with COPD and healthcare providers may contribute to better outcomes in patients who attend our PR programme.

As a compulsory therapeutic intervention, there is a need to provide nutritional supplementation for the patients with COPD.^33^ There is evidence to indicate nutritional supplementation will enhance body weight, increase fat-free mass and body fat density, along with reducing the relapse rate of disease, enhancing tolerance of symptoms and improving the overall quality of life.^33 34^ Since COPD is a pulmonary inflammation, which will need strong innate and adaptive immune support, both micronutrient^35^ and macronutrient replacement will be a mandatory requirement for better recovery. Along with this, it is mandatory to address the nutritional practices and food selections that can contribute to better health. For instance, the reduction in the intake of milk, milk products and eggs is due to the belief that it exacerbates the phlegm and worsens the difficulty in breathing. This phenomenon may result in the emergence of significant nutritional deficiencies among adults with COPD. Pulmonary diseases emerged as one of the most common causes of malnutrition in a hospital-based study in Sri Lanka.

There is evidence to indicate the potential of singing as an adjunct to conventional COPD self-management strategies in high-income countries like the UK.^36^ Singing can elicit a range of psychological and physiological benefits relating to physical capacity, mood, and, when delivered as part of a group, reduce social isolation.^36 37^ Incorporating music into exercise-based interventions, such as PR, can act as a distractive auditory stimulus to reduce sensations of breathlessness and muscle fatigue.^38^ Music and singing have been viewed positively as potential components of effective self-management for individuals with COPD in Uganda, with minimal previous PR exposure.^39^ By integrating culturally familiar elements such as traditional music and songs, healthcare providers can increase patient engagement, compliance and the overall success of rehabilitation programmes.

Through the discussions and interviews conducted in the current study, it was identified that Sri Lankan people who live with COPD and stakeholders preferred to add singing and music components to the PR programme as a cultural adaptation. Singing has physical and psychological effects which can lead to improvements in symptoms and is therefore accepted as an effective method of respiratory exercises in several health settings.^40^ Cultural adaptations to PR have previously been explored in Kyrgyzstan, by adding their traditional dance for a newly developed PR programme,^15^ and in India, where the concepts of yoga exercises were added, for greater acceptability of PR.^41^ By integrating culturally familiar elements such as traditional dance or yoga, healthcare providers can increase patient engagement, compliance and the overall success of rehabilitation programmes.

This study identified several main barriers to the implementation and undertaking of the PR. It includes issues relating to participation, limited availability of medical expertise and difficulties encountered when performing exercises. Occupation became a main barrier for participation as many of them were unable to adjust time away from work and obtain leave to attend the sessions. Numerous studies investigating barriers to PR identified occupation as a significant barrier in the context of PR participation.^42^ Another raised issue was that, since many of the adults must be accompanied by a caregiver to attend the sessions, they too had to obtain leave on these days. This results in two people being away from work on 2 days of the week. Thus, the consistent loss of income on 2 days of the week was not economically viable to the family.

Our findings reveal some differences in components of PR deemed essential in the Sri Lanka context. The psychosocial impacts of COPD on patients suggest the need for psychological support as part of PR, which is supported by work from the Kyrgyz Republic which identified stigma of TB as a significant barrier to accessing healthcare and returning to economic productivity.^15^ Consequently, the psychosocial component of PR in LMICs, including Sri Lanka, appears to be a priority for patients, contrasting with consensus guidelines in high income countries (HICs) which placed psychological assessment as a ‘desirable’ component of PR.^43^ From an LMIC perspective, it may be more appropriate as an ‘essential’ component, as qualitative studies across LMIC involving individuals with chronic respiratory diseases (CRDs)report significant psychosocial issues.

While our findings focus on a traditional centre-based PR, in accordance with previously identified preferences of people living with COPD in Sri Lanka,^23^ non-traditional models of PR (eg, telerehabilitation or home-based PR) may also facilitate addressing identified challenges to establishing PR services. These have been explored in other contexts, such as for those living with COPD or interstitial lung disease (ILD) in India.^44 45^

They face resource limitations in terms of diagnostic tools, rehabilitation equipment and medications necessary for managing chronic respiratory diseases.^46^ Improving the availability of medical resources through capacity building, training programmes and resource sharing programmes, collaborating with the HICs would be beneficial.

The study concentrates on the difficulties encountered in conducting exercises at home, which can be solved by providing suggestions such as use of easy techniques, such as use of bottles filled with water for exercise, to engage in day-to-day activities at home so that exercise capability increases with time. However, many patients with COPD were demotivated to engage in regular exercise.^47^ This pattern is seen in many Asian settings where the patient is well cared for at home so that he does not have to engage in any form of physical activity.

## Strengths and limitations

This study was strengthened through the inclusion of a wide variety of stakeholders and a diverse population of Colombo city. However, it was limited to the CCC in Colombo; thus, the findings may not reflect the ideas and perceptions of patients with COPD in other districts in Sri Lanka. Also, since the data collection was done during the COVID-19 pandemic, there was a limitation on the access of caregivers as CCC had made restrictions on visitors.

## Conclusion

Since many healthcare settings in Sri Lanka do not have an established PR programme, it is imperative to implement this low-cost, high-impact intervention to improve the health and well-being of people living with COPD. The requirement of culturally specific PR was highlighted in this study, with specific needs to tailor the educational component for service users and their caregivers, adding appropriate nutritional supplementation and singing and music as a culturally acceptable activity to enhance compliance among people living with COPD. Addressing these identified barriers would facilitate the implementation of PR in Sri Lanka and support patient compliance to the course. The results of this study can be adopted by existing PR programmes in Sri Lanka to deliver a culturally acceptable programme.

## Supplementary material

10.1136/bmjresp-2024-002407 online supplemental file 1

## Data Availability

No data are available.
