# Supplementary material for: How to culturally adapt the pulmonary rehabilitation programme for people living with COPD in Sri Lanka: a qualitative study
Source: BMJ Open Respir Res. 2025 Jul 21;12(1):e002407. doi: 10.1136/bmjresp-2024-002407 (PMC12281325; doi:10.1136/bmjresp-2024-002407)
Supplement: online supplemental file 1 [file bmjresp-12-1-s001.pdf]

Annexure 1.1

Work Package 01

Focus group guidelines - Patients with COPD

- **Aim:** To explore the care seeking behaviors, perceived needs and their desires to improve self- management of COPD as perceived by adults with COPD, in selected healthcare settings in Sri Lanka.

1. Please explain what "Chronic obstructive pulmonary disease" means to you?
2. Please tell me what is your experience of having COPD.
    a. When were you first diagnosed?
    b. How did you expect COPD was going to affect you?
    c. What impact has COPD had on your life? Prompt: (employment), your domestic tasks, and leisure choices). What has been the most significant? What? It what ways? Give examples.
    d. How would you describe COPD to someone who has never heard it before?
    e. Why do you think you have COPD? What caused it?
    f. Did you know anyone else who had COPD? How has it affected them?
3. In your perspectives what are your suggestions to control your COPD (e.g.,? breathing difficulty)?
4. What are your thoughts regarding PR/ Exercise programme?
5. What kind of advice have you received regarding pulmonary rehabilitation?
    a. If so, what? Why? How would this have helped you? (Prompts: What is it? Lungs? Activity? Diet? Symptoms? Medication? Causes?)

   b. How would you like this information to look? How should it be made available to people? (Prompts: paper? Online?)

6. What are the obstacles that you have faced when following instructions in pulmonary rehabilitation? Please explain.

   Probing questions : How this COVID pandemic is effecting on participating on PR programme? Has it been a problem for you, or will it be a problem?

   Prompt: transport, support to attend?

7. What would you expect to happen on the PR programme?
8. As you think what type of exercises and educational sessions will be more usefull for you?
9. How would you hope PR might help you? What do you hope to achieve from taking part? What would help?
10. what sort of help do you need to manage your COPD condition ? (PROMPT, type of support, education, information, types of exercises, nutritional needs)
11. Are the any activities or hobbies that could be useful in PR? (PROMPT: cultural adaptations)

Probing questions:

What types of physical activity would you be happy doing more of? What would you not be happy doing more of? Is this level of activity your choice or are you forced to be this active?

Do you prefer individual or social activities when you are being active? Why?

What kind of activities do you see other people doing that you can't do? What kind of activities do you see other people doing that you would like to do?

What kind of activities would fit in with your life or be too inconvenient? This might be in terms of time or being with other people. What activities could you do with others or would want to do with others? What kind of activities would you want to do alone?

What kind of activities would you expect PR to include? (Prompt: cultural adaptations to PR) What would you not want to do as part of this?

What are the nutritional beliefs that you are having in concerning of your disease condition? Please explain.

Thank you for your participation!

Annexure 1.2

Work Package 01

Relations of patients with COPD – Focus group discussion guidelines

Aim: The main aim of this discussion is to explore the idea of patient's relations/ care takers regarding the impact of their relator's disease condition on family functions and idea on improving physical activity level and promoting pulmonary rehabilitation

1. How did you react when you were told your family member has COPD? How did you feel?
2. How do you help your family member with their condition?
3. What has been the most significant change for your family member?
4. In regards to the physical activity what do you think about your caretaker's/ relation's day to day physical activity?
5. According to your perspective, what will be the most effective ways in improving physical activity of your caretaker's/ relation's?
6. As you feel, what will be the most possible and beneficial method and time in delivering PR/ Exercise programme for your relation?
7. As you think what will be the barriers and obstacles you may face when your caretaker's/ relation's are following PR/ Exercise programme? (Prompt: COVID – safety considerations).
8. Is there anything you wish you had been told about COPD in order to help care for your family member? If so, what? Why? How would this have helped you? (Prompts: What is

it? Lungs? Activity? Diet? Symptoms? Medication? Causes?). How would you like this information to look? How should it be made available to people? (Prompts: paper? Online?)

9. What do you think should be included in PR? Is there any activities your family member might enjoy? Any activities which might help them?
10. Do you have any suggestions in regards to this Exercise programme ?

11. Do you think relations/caregivers should be involved during PR? How? Why?

Probing questions:

Could you please tell us something about your nutrition related practices especially regards to the COPD on your caretaker's/ relation's?

Thank you very much for your participation.

Annexure 1.3

Focus group guidelines – Nurses

Aim: The study aimed to explore the needs and perceptions of health care professionals to inform the adaptations required for a PR programme, suitable for the Sri Lankan context and envisaged outcomes of the focus groups discussions will inform the design and content of Sri Lankan specific PR

1. Briefly introduce yourself. Tell us about your experience on caring for patients with COPD?

   - Prompt: Number of years of experience with COPD patients

2. Could you please, brief us regarding your experience on PR and What does pulmonary rehabilitation mean to you?

   - Prompt: Number of years of experience involved in PR
   - Prompt: What is your role in PR

3. How important do you think it is to provide PR?

   - Prompt: Why do you think this?
   - Prompt: What do you hope patients would get from participating in PR?
   - Prompt: What should be the priorities for PR to help patients with?
   - Prompt: What are patients most in need of help of?

5. How do you think patients will get on with PR?

6. What are the challenges for patients with COPD participating in PR?

   - Prompt: Why do you think this?

7. How do you think these challenges can be resolved?

   - Prompt: Why do you think this?

8. What do you think should be included in PR?

   - Prompt: education, information, types of exercises, nutritional needs

- Prompt: Why do you think this?
- What would help to make PR more appealing to patients?

9. Are the any activities or hobbies that could be useful for patients in PR?

- Prompt: Cultural adaptations, Singing and dancing, meditation/ mindfulness
- Prompt: Why do you think this?

10. What are the adaptions you recommend for the PR during COVID pandemic

- Prompt: COVID (safety, handwashing, group based activities, social distancing, face masks)
- Probing question: Location – How do you think patients will feel about going to Central Chest Clinic for PR? Prompt: Group setting, hospital setting
- Number of maximum patients can be accommodated for PR usually and during pandemic.

11. Do you have any suggestions to improve/recommendations in regards to the PR among COPD patients?

Thank you for your participation!

Annexure 1.4

Semi Structured Questions guidelines – Physiotherapist

Aim: The study aimed to explore the needs and perceptions of health care professionals to inform the adaptations required for a PR programme, suitable for the Sri Lankan context and envisaged outcomes of the semi structured interviews will inform the design and content of Sri Lankan specific PR

1. Briefly introduce yourself. Tell us about your experience on patient with COPD?

   - Number of years of experience with COPD

2. Could you please, brief us regarding your experience on PR and What does pulmonary rehabilitation mean to you?

   - Number of years of experience involved in PR
   - What is your role in PR

3. How important do you think it is to provide PR?

   - Prompt: Why do you think this?
   - Prompt: What do you hope patients would get from participating in PR?
   - Prompt: What should be the priorities for PR to help patients with?
   - Prompt: What are patients most in need of help of?

5. How do you think patients will get on with PR?

6. What do you think should be included in PR?

   - Prompt: education, information, types of exercises, nutritional needs
   - Prompt: Why do you think this?
   - What would help to make PR more appealing to patients?

7. Are the any activities or hobbies that could be useful for patients in PR?

   - Prompt: Cultural adaptations, Singing and dancing, meditation/ mindfulness
   - Prompt: Why do you think this?

8. What are the adaptions you recommend for the PR during COVID pandemic

- Prompt: COVID (safety, handwashing, group based activities, social distancing, face masks)
- Probing question: Location – How do you think patients will feel about going to Central Chest Clinic for PR? Prompt: Group setting, hospital setting
- Number of maximum patients can be accommodated for PR usually and during pandemic.

9. Do you have any suggestions to improve/recommendations in regards to the PR among COPD patients?

Thank you for your participation!

Annexure 1.5

<u>Semi Structured Questions guidelines - Doctors</u>

Aim: The study aimed to explore the needs and perceptions of health care professionals to inform the adaptations required for a PR programme, suitable for the Sri Lankan context and envisaged outcomes of the semi structured interviews will inform the design and content of Sri-Lankan specific PR

1. Briefly introduce yourself. Tell us about your experience on management of COPD patients?

    - Prompt: Number of years of experience with COPD

3. What does rehabilitation mean to you?

4. How important do you think it is to provide PR?

    - Prompt: Why do you think this?

5. How would do you describe PR to patients?

6. For what reasons would you would you refer patients to PR?

    - What symptoms?

7. How do you think patients will respond to your referral for PR?

    - Prompt: Why do you think this?

8. What do you think the challenges are when referring patients to PR? How do you think these can be resolved?

9. How do you think patients will get on with PR?

    - Prompt: Why, can you explain?

12. What do you think should be included in PR?
    - Prompt: education, information, types of exercises, nutritional needs
    - Prompt: Why do you think this?

13. Are the any activities or hobbies that could be useful for patients in PR?
    - Prompt: Cultural adaptations, Singing and dancing, meditation/ mindfulness
    - Prompt: Why do you think this?

10. What will be the challenges for patients with COPD participating in PR?

11. How do you think these challenges can be resolved?
    - Prompt: COVID (safety, handwashing, group based activities, social distancing, face masks)

14. Do you have any suggestions to improve/recommendations in regards to the PR among COPD patients?

Probing questions:

1. When considering about the physical activity, what will be the most effective ways in improving physical activity among patients with COPD? Please explain.
2. According to your experience, please explain obstacles/barriers for patients when they are improving physical activity.
3. Regarding the nutritional improvement, please explain useful methods to improving nutritional status of the patients with COPD?
4. According to your experience, please explain obstacles / barriers for patients when they are improving nutritional status.

Thank you for your participation!
